# Mechanistically derived fluorescence rancidity index for real-time monitoring of olive oil oxidation under thermal, photo, and frying conditions

**DOI:** 10.1016/j.fochx.2026.104381

**Published:** 2026-09-01

**Authors:** Yubeen Lee, Jee Won Lee, Jinhee Kang, Won Young Jeong, Hyung Min Kim, Jee-Young Imm

**Affiliations:** a Department of Foods and Nutrition, Kookmin University, Seoul 02707, Republic of Korea; b Department of Applied Chemistry, Kookmin University, Seoul 02707, Republic of Korea

**Keywords:** Fluorescence spectroscopy, Olive oil, Lipid oxidation, Chlorophyll, Fluorescence rancidity index (FRI)

## Abstract

A fluorescence-based method was developed for rapid, non-destructive monitoring of olive oil oxidation. The fluorescence rancidity index (FRI) was introduced as a ratio-based optical indicator normalized to fresh oil fluorescence. Using a custom-built laser-induced fluorescence system (532 nm excitation), extra virgin and pomace olive oils were evaluated under thermal oxidation (180 °C), UV oxidation, and repeated frying. Oxidation caused progressive attenuation and a blue shift of chlorophyll-associated emission (∼675 nm). Under thermal oxidation, FRI showed strong correlations with PAV (*r* = 0.92), TOTOX (*r* = 0.91), INTOX (*r* = 0.92), and CDV (*r* = 0.88), whereas no significant correlations were observed under UV oxidation. FRI also increased progressively during repeated frying. These findings support FRI as a rapid, reagent-free optical indicator of olive oil oxidation, particularly under thermal conditions, while highlighting its oxidation-pathway-dependent response.

## 1 Introduction

Olive oil, especially extra virgin olive oil (EVOO), is a fundamental component of the Mediterranean diet and is recognized for its exceptional sensory qualities and health- promoting effects. These qualities are primarily attributed to abundant oleic acid, pigments, and phenolic compounds, which contribute to both flavor and oxidative stability (Santos et al., 2013). Specifically, the health benefits of extra virgin olive oil (EVOO) are largely attributed to its phenolic constituents, including hydroxytyrosol, tyrosol, and oleuropein. Among these, hydroxytyrosol particularly notable for its cardioprotective effects, achieved by improving endothelial function and inhibiting low-density lipoproteins (LDL) oxidation, thus reducing atherogenic oxidized LDL formation (Bulotta et al., 2014; Tejada et al., 2017). In addition to these biological activities, phenolic compounds, and chlorophyll pigments, significantly enhance the oxidative stability of olive oil by scavenging reactive oxygen species and suppressing lipid peroxidation. The oxidative degradation of these endogenous antioxidants directly modifies the fluorescence characteristics of olive oil, providing the mechanistic basis for the fluorescence-based monitoring approach proposed in this study.

Lipid oxidation during thermal processing or extended storage is a principal factor in quality deterioration, leading to the loss of nutritional value and the formation of volatile and polymeric oxidation products that impair sensory characteristics (Choe & Min, 2006).

Conventional analytical methods for assessing oil oxidation include peroxide value (POV), *p-*anisidine value (PAV), conjugated diene value (CDV), conjugated triene value (CTV), and thiobarbituric acid-reactive substances (TBARS) (Abeyrathne et al., 2021). Despite their widespread use, these wet-chemical methods have inherent drawbacks, such as matrix interferences, variability in reaction conditions, and limited sensitivity to early oxidation stages (Barriuso et al., 2013; Kiokias et al., 2010). Consequently, multiple methods are often combined to adequately characterize the complexity of lipid oxidation processes. Recent advances in analytical chemistry have emphasized spectroscopic techniques as rapid, non-destructive alternatives for food quality monitoring. Among these, fluorescence spectroscopy stands out for its high sensitivity, minimal sample preparation, and strong signal-to-noise ratio (Fang et al., 2019).

EVOO contains natural fluorophores, including chlorophylls, tocopherols, and oxidation-derived compounds, which emit characteristic fluorescence signals that evolve during oxidation (Jung et al., 2011; Sikorska et al., 2012). Chlorophyll is especially relevant to oxidative stability because it can act as a photosensitizer under light exposure and, during thermal or photo-oxidative stress, its degradation accompanies pigment bleaching and accelerated oxidation, making it a useful endogenous indicator of oil deterioration (Balaky et al., 2020).

Previous research has demonstrated that fluorescence spectroscopy effectively monitors EVOO oxidation kinetics and correlates well with traditional parameters such as POV and UV absorbance at 232 nm (K232) (Mishra et al., 2018). Furthermore, excitation-emission matrix (EEM) fluorescence combined with chemometric tools or machine learning, has enabled the quantitative prediction of oxidation-related indices (*r*^*2*^ > 0.9) and development of portable tools to support the quality assessment of EVOO (Martin-Tornero et al., 2022; Venturini et al., 2024).

Although previous studies have established fluorescence spectroscopy as a promising tool for monitoring olive oil oxidation, quantitative interpretation has often depended on individual spectral features or multivariate models. In the present study, we introduced a fluorescence rancidity index (FRI) that converts the decrease in chlorophyll-associated fluorescence into a normalized optical index using the fluorescence of fresh oil as a reference. The FRI is calculated as the decrease in the integrated fluorescence intensity of the chlorophyll-associated region (640–725 nm) in oxidized oil relative to the initial integrated intensity of fresh oil. Rather than analyzing chlorophyll loss and oxidation-associated fluorescence change, the FRI combines their evolution into a single normalized parameter. This ratio-based approach reduces sensitivity to absolute fluorescence intensity and facilitates comparison of oxidation-dependent spectral changes among the tested oils. Furthermore, the FRI can be calculated directly from fluorescence spectra without reagent-based chemical analysis or chemometric model training, offering a simple and rapid method for optical monitoring of olive oil extraction.

A laser-induced fluorescence (LIF) technique was developed for the in situ assessment of olive oil oxidation. The method was designed to: (i) optimize excitation and emission parameters for enhanced sensitivity to oxidation products; (ii) integrate chemometric modeling to predict conventional oxidation indices (POV, PAV, CDV, CTV); and (iii) evaluate performance under thermal- and photo-oxidative conditions. Comparative analysis with standard methods demonstrates that LIF provides a rapid, reagent-free, non-destructive, and predictive platform for monitoring olive oil oxidative stability.

## 2 Materials and methods

### 2.1 Materials

Commercially available olive oils (EVOO and pomace olive oil [POO]) were purchased from a local supermarket in Seoul, Korea. Glacial acetic acid (>99.7%), hexane (>99.5%), and *p-*anisidine (>99%) were obtained from Sigma-Aldrich (St. Louis, MO, USA). Dichloromethane and 2,2,4-trimethylpentane (isooctane) were supplied by JUNSEI (Tokyo, Japan). Potassium iodide (KI >99%) was procured from Samchun Pure Chemical Co. (Seoul, Korea). Starch indicator solution (1%, *w*/*v*) was obtained from Alfa Aesar (Ward Hill, MA, USA), and sodium thiosulfate pentahydrate was sourced from Yakuri Pure Chemicals (Kyoto, Japan).

### 2.2 Preparation of samples

#### 2.2.1 Thermal oxidation

Each oil sample (A, B, and C: EVOO; D: POO; 3 L each) was subjected to controlled thermal oxidation in an electric deep fryer (Model DK-301, Daekwang, Korea) maintained at 180 ± 2 °C. This temperature was selected to simulate conventional frying conditions (Chen et al., 2025). Heating durations were set at 0, 4, 8, and 16 h to monitor progressive oxidation under prolonged thermal exposure. After each interval, approximately 50 g of oil was collected and stored at −24 °C until chemical analysis.

#### 2.2.2 UV oxidation

For photo-oxidation studies, 500 mL of each oil sample was placed in transparent glass bottles. Lids were replaced with plastic wrap to allow UV transmission. A UV-B lamp (LAB24, Dongseo Science, Dangjin, Korea) was positioned 20 cm above the samples, delivering approximately 39.2 W of irradiation. Samples (15 g) were withdrawn at 24 h intervals for up to 96 h, then immediately transferred into amber vials and stored at −24 °C until analysis.

#### 2.2.3 Simulated frying experiment

A simulated frying experiment was conducted to replicate practical culinary usage conditions. Pre-frozen potato strips (150 g per batch) were fried at 180 °C for 3 min in a temperature-controlled fryer. Oil samples were collected after 5, 10, 15, and 20 consecutive frying cycles, without oil replenishment, to simulate cumulative degradation typical of commercial frying operations. Following collection, samples were cooled, filtered through a 0.45 μm PTFE membrane to remove food particles, and stored at −24 °C in the dark until analysis.

### 2.3 Fluorescence measurement setup

**Fig. 1:**
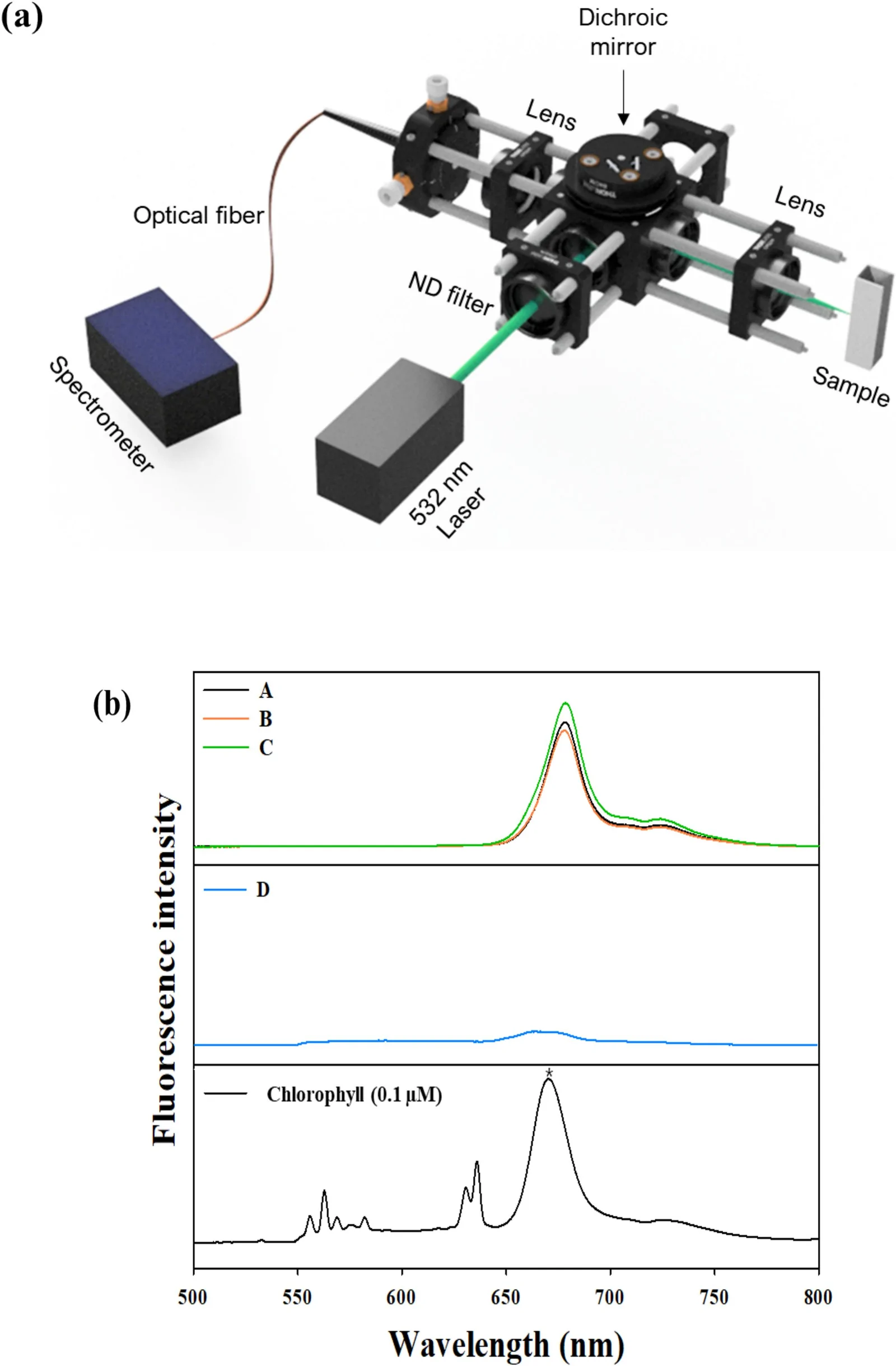
**(a)** Schematic configuration of the fluorescence measurement system employing a 532 nm laser excitation source. **(b)** Fluorescence emission spectra of olive oil samples and chlorophyll *a* in toluene (0.1 μM). Samples A-C represent extra virgin olive oils (EVOO), and sample D represents pomace olive oil (POO).

A custom-built laser-induced fluorescence (LIF) spectroscopy system was developed for real-time monitoring of olive oil oxidation (Fig. 1a). The system comprised three main components: an excitation pathway, a sample compartment, and a fluorescence collection pathway. A 532 nm diode laser (CNI Optoelectronics Tech., China) served as the excitation source, chosen for its efficient excitation of chlorophyll-associated pigments in olive oil and compatibility with compact optics. Laser intensity was controlled using a neutral-density filter, and the excitation beam was directed to the sample via a dichroic mirror, then focused with a lens. Olive oil samples (3 mL) were placed in cuvettes mounted in a holder to ensure consistent optical alignment, with the excitation beam focused at a distance of 30 mm. Backward-emitted fluorescence was collected along the same optical axis, passed through a long-pass filter to suppress backscattered 532 nm excitation light, and coupled into an optical fiber. The fluorescence signal was transmitted to a spectrometer (SM303, Korea Spectral Products, Korea) operating over 300–800 nm. Spectra were acquired using a 0.1 s- integration time, and each measurement was repeated three times to assesses reproducibility.

To demonstrate the practical applicability of the proposed approach, a compact LIF prototype was additionally developed (**Fig. S2**). Although fluorescence spectra in this study were acquired using a laboratory spectrometer, the prototype demonstrates the feasibility of integrating the essential excitation and detection components into a portable platform for in situ measurements. This configuration enables rapid, non-destructive fluorescence analysis of olive oil under diverse oxidative conditions without the need for reagent-based sample preparation.

### 2.4 Conventional lipid oxidation analyses

#### 2.4.1 POV and PAV

POV, which quantifies the primary oxidation products (lipid hydroperoxides), was determined according to the AOCS official method Cd 8–53 (AOCS, 2017a). Oil samples (5 g) were dissolved in 30 mL of glacial acetic acid-dichloromethane (3:2, *v*/v). Then, 0.5 mL of saturated potassium iodide (KI) solution was added to induce the stoichiometric liberation of iodine ($I_{2}$) by the hydroperoxides present in the sample. The liberated iodine was subsequently titrated with a standardized 0.01 M sodium thiosulfate ($Na_{2}S_{2}O_{3}$) solution using a 1% (*w*/*v*) starch solution as the indicator. The POV was calculated using the following equation:

(1) $$\mathrm{POV}\;\left(\mathrm{meq}/g\right)=\frac{\left(V\times N\right)\times 1000}{m}$$

where, *V* is the volume (mL) of sodium thiosulfate consumed, *N* is its normality, and *m* is the sample weight (g).

PAV, an indicator of secondary oxidation products, was determined according to the AOCS official method Cd 18–90 (AOCS, 2017b). Each oil sample (0.1 g) was diluted in 10 mL of isooctane. An aliquot (5 mL) of each solution was mixed with 1 mL of 0.25% (*w*/*v*) *p-*anisidine solution prepared in acetic acid. After incubation in the dark for 10 min to allow the formation of chromophores via the condensation reaction between the carbonyl groups of aldehydes and the amino group of p-anisidine. The absorbance was measured at 350 nm using a UV–visible spectrophotometer. PAV was calculated as:

(2) $$\mathrm{PAV}=\frac{25\times \left(1.2\;\mathrm{As}-\mathrm{Ab}\right)\;}{m}$$

where, *As* and *Ab* represent absorbance values after and before reaction with *p-*anisidine reagent, respectively, and m is the sample mass (g).

#### 2.4.2 Total oxidation (TOTOX) and integrated oxidation (INTOX) indices

Total oxidation (TOTOX) and integrated oxidation (INTOX) indices were calculated to evaluate the combined presence of primary and secondary oxidation products. TOTOX which emphasizes the initial state of oxidation, was determined using Eq. (3):

(3) $$\mathrm{TOTOX}=\left(2\times \mathrm{POV}\right)+\mathrm{PAV}$$

The INTOX index, which offers a higher weight to the stable secondary degradation products and provides superior sensitivity during advanced thermal oxidation stages, was calculated as per Eq. (4):

(4) $$\mathrm{INTOX}=\mathrm{POV}+\left(2\times \mathrm{PAV}\right)$$

These complementary indices provide a more comprehensive assessment of lipid oxidation than POV or PAV alone by accounting for both hydroperoxide formation and the accumulation of stable carbonyl compounds during oxidation.

#### 2.4.3 CDV and CTV

CDV and CTV were determined to evaluate structural changes in unsaturated fatty acids during lipid oxidation. CDV and CTV were measured according to the methods described by Saguy et al. (1996) and Qianchun et al. (2014), respectively. Oil samples were diluted 600-fold in hexane and agitated for 3 h. Hexane was used as the blank, and absorbance was measured at 234 nm for CDV and 270 nm for CTV using a UV–visible spectrophotometer (Ultrospec 2100 Pro, Amersham Biosciences, Uppsala, Sweden). The concentration of conjugated dienes was calculated using a molar extinction coefficient of 29,000 M/cm.

### 2.5 Statistical analysis

Data are expressed as the mean ± standard deviation (SD). One-way analysis of variance (ANOVA) was performed to evaluate significant differences among samples (*P* < 0.05). When significant differences were observed, Duncan's multiple range test was used for *post-hoc* comparisons. The statistical analyses were conducted using SPSS 26.0 software (SPSS, Inc., Chicago, IL, USA). Pearson correlation coefficients (r), two-sided *p*-values, and 95% confidence intervals (CIs) were calculated to assess associations between the FRI and conventional oxidation indices, including POV, PAV, TOTOX, INTOX, CDV, and CTV. Correlation analyses were performed separately for thermal and UV oxidation, each comprising 16 oil–time condition (four oil samples × four time points). Triplicate measurements for each oil–time condition were averaged before statistical analysis. Simple linear regression was also performed to characterize the relationship between FRI and each oxidation index, with slope, intercept, and coefficient of determination (R^2^) reported (**Table S1**). All statistical analyses were performed using MATLAB R2024a (MathWorks, Natick, MA, USA).

## 3 Results and discussion

### 3.1 Fluorescence spectra of olive oil and chlorophyll

Fluorescence spectra of EVOO, POO, and a chlorophyll standard solution were acquired using the custom-built LIF system (Fig. 1a). All measurements were conducted under identical conditions using a 532 nm excitation source, with emission spectra collected from 300 to 800 nm. Both EVOO and POO exhibited fluorescence profiles closely resembling the chlorophyll standard, confirming that chlorophyll *a*nd chlorophyll derivatives are the dominant fluorophores in fresh olive oils (Fig. 1b)**.** The prominent emission band centered at approximately 675 nm is attributed to chlorophyll *a* (Chl*a*) and its derivatives, which are the primary pigments retained during the oil extraction process (Gandul-Rojas et al., 2016).

The fluorescence intensity of the chlorophyll standard solution (prepared in toluene) increased linearly with concentration (0.01–0.08 μM; **Fig. S1**), demonstrating a robust quantitative relationship. This supports the suitability of fluorescence spectroscopy for the reliable detection and quantification of chlorophyll in olive oils (Borello & Domenici, 2019). Because toluene exhibits Raman scattering under 532 nm laser excitation, solvent background subtraction was applied to eliminate Raman-related features. This correction resulted in minor spectral artifacts, indicated by an asterisk in Fig. 1b, but ensured that the recorded fluorescence signals originated exclusively from pigment emission rather than solvent-induced interference.

### 3.2 Fluorescence rancidity index (FRI)

Lipid oxidation in edible oils is a complex, radical-driven chain reaction initiated by the formation of reactive oxygen species (ROS), hydroperoxides, and free fatty acids. These oxidative species not only deteriorate sensory and nutritional quality but also induce structural and chemical modifications in endogenous pigments, particularly chlorophylls and their derivatives (Gandul-Rojas et al., 2016). Because chlorophylls and chlorophyll-derived pigments are intrinsic fluorophores in olive oil, changes in their composition and structure can be reflected in the fluorescence characteristics of the oil. Previous studies have shown that thermal degradation of chlorophyll-related pigments in virgin olive oil involves pheophytinization-replacement of, the central Mg^2+^ ion in the chlorophyll porphyrin ring by two H^+^ ions, followed by further transformations that can generate pyropheophytins and other degradation products (Aparicio-Ruiz et al., 2010; Li et al., 2015). These structural transformations alter the photophysical properties of chlorophyll-derived pigments and explain the progressive attenuation and spectral shift of chlorophyll-associated fluorescence observed in the present study. Thus, the decrease in chlorophyll-associated fluorescence serve as a rapid optical indicator that can be monitored alongside lipid oxidation. However, because individual chlorophyll degradation products were not directly identified or quantified in this study, these pigment transformations should be considered a literature-supported interpretation of the observed fluorescence changes, rather than a directly demonstrated reaction pathway.

Additionally, the spectra revealed the formation of fluorescent oxidation products, including carbonyl compounds and conjugated double-bond structures, as evidenced by the increase fluorescence in the short-wavelength region. Along with the progression of lipid oxidation, the chlorophyll-associated fluorescence progressively decreased. To quantify this spectral change, the fluorescence intensity was integrated over the chlorophyll-associated emission region of 640–725 nm, representing chlorophyll-associated fluorescence that progressively decreased. This spectral response forms the basis of a ratio-based fluorescence indicator for oxidation. For each fluorescence spectrum, integrated intensity was calculated over 640–725 nm, chlorophyll-associated fluorescence decreasing with oxidation.

The fluorescence ratio (**I**) was defined as:

(5) $$I=\int_{640}^{725}F\left(\lambda \right)\;\mathrm{d\lambda}$$

where $F\left(\lambda \right)\;$denotes the fluorescence intensity at wavelength λ.

The FRI was then calculated as:

(6) $$\mathrm{FRI}=\frac{I_{\mathrm{fresh}}-I_{\mathrm{sample}}}{I_{\mathrm{fresh}}}$$

where *I*_*fresh*_ and *I*_*sample*_ represent the integrated fluorescence intensities for fresh and oxidized oils, respectively. As oxidation progresses, chlorophyll-associated fluorescence decreases causing the FRI to increase. The same FRI definition and calculation procedure were applied across all thermal, UV-oxidation, and frying experiments.

Similar spectral changes during lipid oxidation have been reported previously. Mishra et al. (2018) observed a marked decrease in chlorophyll emission near 671 nm, accompanied by increased fluorescence in the 420–515 nm range due to the formation of secondary oxidation products. In addition, heat-induced conversion of Chla to pyropheophytin has been reported to cause a blue shift of the emission maximum to around 665 nm (Kongbonga et al., 2015).

The proposed FRI demonstrated consistent, quantitative, and reproducible trends across all oxidative conditions. Its spectrally normalized formulation enables direct comparison across different oil samples independent of absolute intensity variations, facilitating a standardized assessment of oil rancidity. This feature is particularly advantageous for portable or in situ applications, where rigorous instrument calibration may be impractical. From a practical perspective, the FRI represents a robust, *non-destructive optical indicator* for real-time monitoring of olive oil quality and oxidative stability, offering a reagent-free complement or alternative to conventional titration-based oxidation analyses.

### 3.3 Thermal oxidation of olive oil

**Fig. 2:**
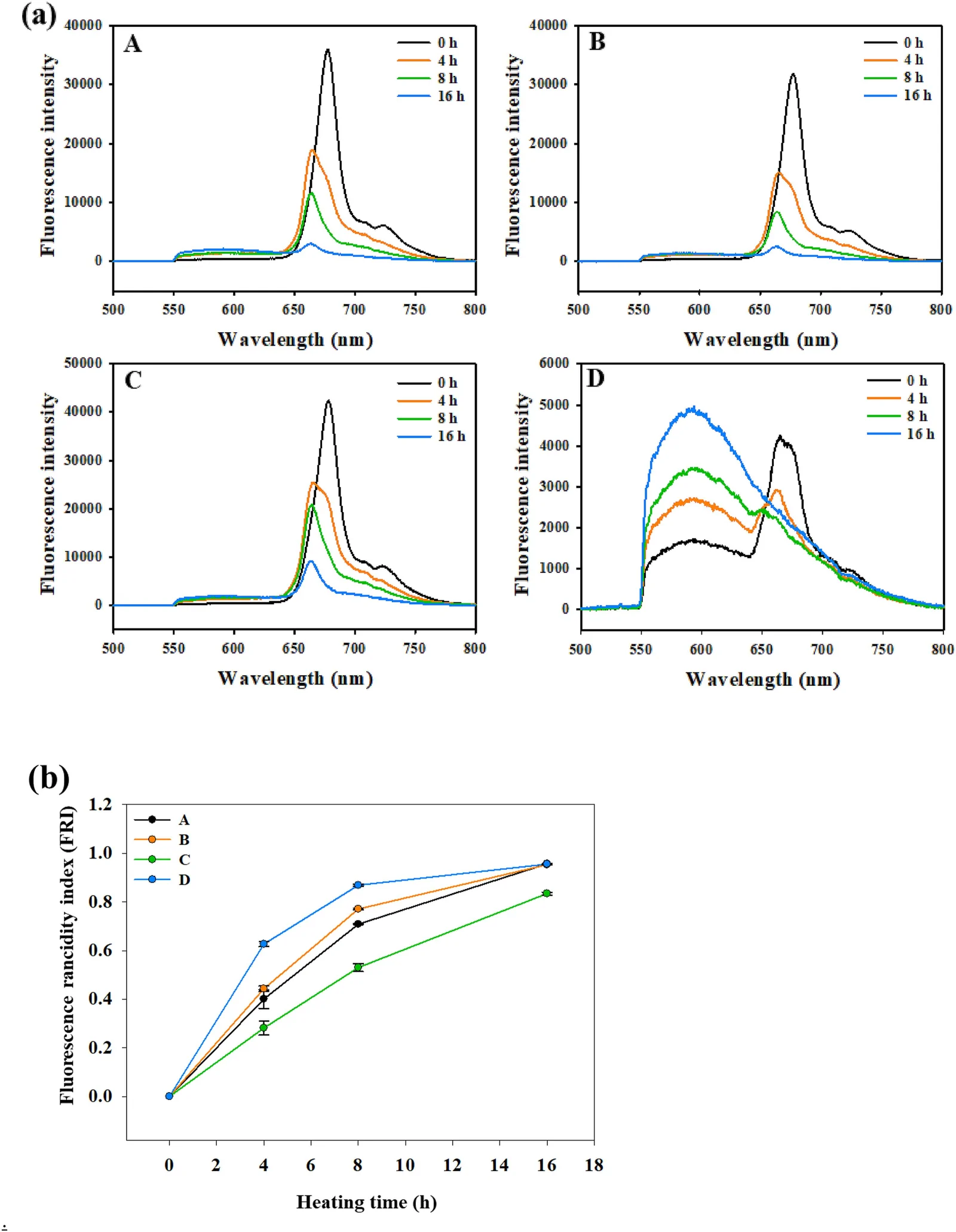
**(a)** Evolution of fluorescence spectra of olive oil samples heated at 180 °C for 0, 4, 8, and 16 h. **(b)** Time-dependent changes in fluorescence rancidity index (FRI) values calculated from the relative decrease in integrated fluorescence intensity over 640–725 nm. Error bars indicate the standard deviation (SD) of triplicate measurements.

Fig. 2a illustrates the evolution of fluorescence spectra for the four olive oil samples subjected to thermal treatment at 180 °C for up to 16 h. A progressive decrease in the Chl*a* emission peak (∼ 675 nm) was observed across all samples as heating time increased. Unlike the Chl*a* standard solution, which exhibited only an intensity reduction, the olive oil samples showed an additional gradual blue shift toward shorter wavelengths. This spectral shift suggests that thermal oxidation of olive oil involves both pigment degradation and chemical transformations that alter the local microenvironment through the formation of primary and secondary oxidation products (Tena et al., 2009).

The progressive degradation and intensity reduction of Chla are intrinsically linked to the chemical events of lipid oxidation. During thermal treatment at 180 °C, the rapid homolytic cleavage of lipid hydroperoxides (primary oxidation products) generates highly reactive lipid peroxyl ($\mathrm{RO}O^{∙}$) and alkoxyl ($RO^{∙}$) radicals. These radical species abstract hydrogen atoms from or directly attack the conjugated porphyrin ring of chlorophyll molecules, inducing oxidative bleaching and accelerating the demetallation process. Consequently, the decrease in chlorophyll fluorescence intensity does not merely reflect thermal decomposition of the pigment alone, but serves as a direct, synchronized reflection of the radical-mediated propagation phase of lipid oxidation, thereby establishing the FRI as a mechanistically grounded optical indicator.

The observed decrease and blue shift in chlorophyll-associated emission are consistent with literature-reported transformations of chlorophyll-related pigment during heating, including changes related to pheophytin and pyropheophytin (Li et al., 2015). These transformations may involve Mg^2+^ removal from the porphyrin ring and side-chain decarboxylation, which can modify the photophysical properties of chlorophyll-derived pigments. However, the present study did not directly identify or quantify these pigment species. The structural conversion of Chl *a/b* into pheophytin *a/b,* and subsequently into pyropheophytin *a/b,* induces fluorescence quenching and wavelength shifts (Li et al., 2015). These transitions occur via Mg^2+^ removal from the porphyrin ring and side-chain decarboxylation, modifying the pigments' photophysical properties. Sample D, (refined POO) displayed markedly lower initial fluorescence intensity than EVOO samples (A-C). This is attributed to the refining process, which removes endogenous pigments and antioxidants including chlorophylls, carotenoids, and phenolic compounds, thereby increasing susceptibility to thermal oxidation (Sikorska et al., 2012).

During heating, the fluorescence spectra exhibited an increase in the short-wavelength region below 638 nm and a progressive decrease in the long-wavelength region around 675 nm. The increased short-wavelength fluorescence is associated with the accumulation of oxidation-derived compounds, such as carbonyl species and conjugated double-bond systems, whereas the decrease in the long-wavelength region reflects chlorophyll degradation (Ansar et al., 2023; Hafeez et al., 2025**)**. For quantitative evaluation, the decrease in the long-wavelength chlorophyll-associated region was used to calculate the FRI. The fluorescence intensity was integrated over the long-wavelength chlorophyll-associated region (640–725 nm). The FRI was then calculated as the difference between the integrated intensity at each heating stage and the initial integrated intensity of fresh oil, normalized to the initial value used as the reference.

All samples exhibited a monotonic increase in FRI values over the heating period (Fig. 2b). Sample D showed the steepest FRI slope over the heating period, indicating the highest oxidation rate, whereas sample C displayed the most gradual increase, reflecting superior thermal stability**.** As oxidation progressed, the FRI values asymptotically approached 1, reflecting near-complete fluorescence quenching and extensive chlorophyll degradation.

**Fig. 3:**
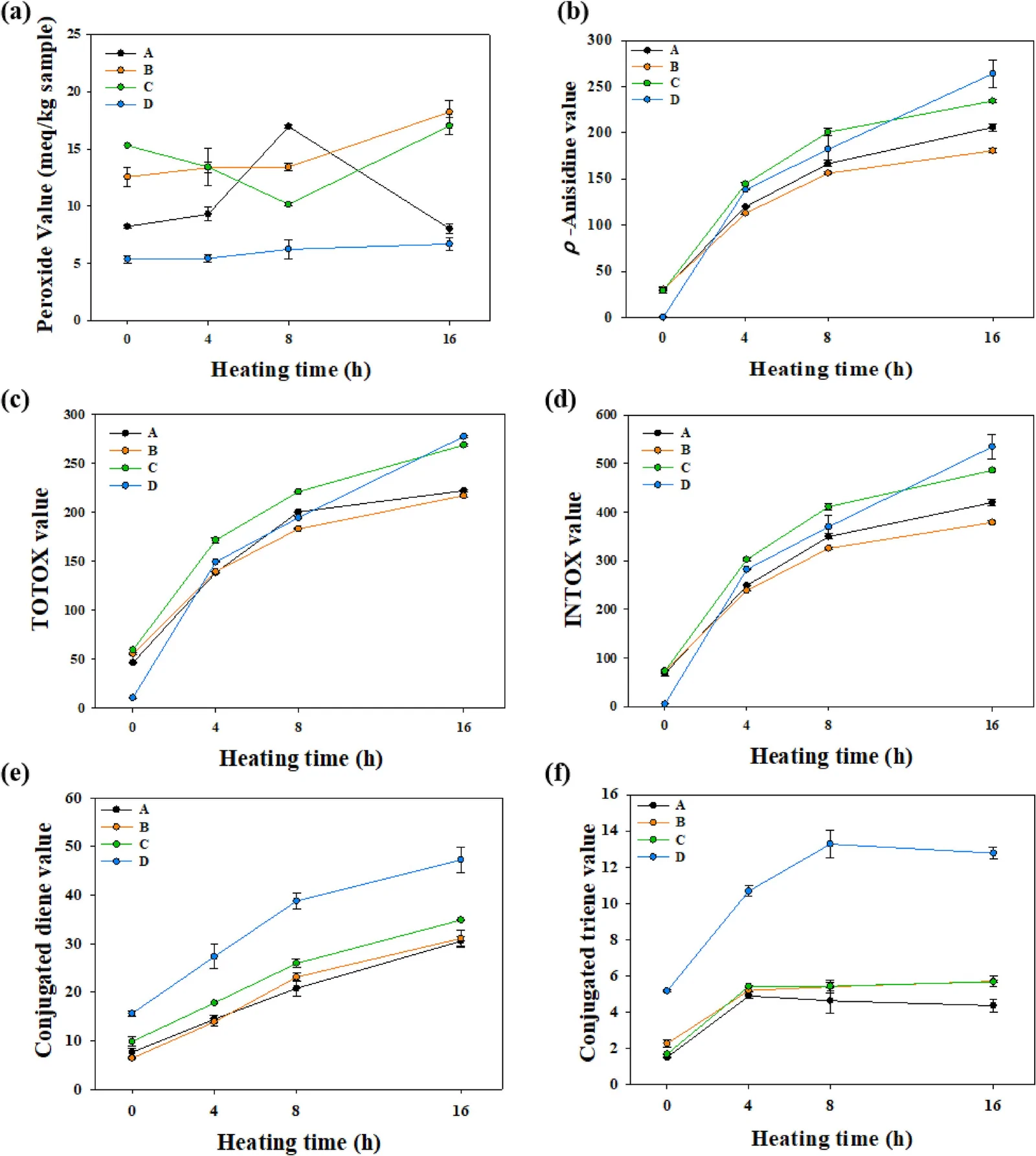
Changes in conventional oxidation indices of olive oil samples heated at 180 °C: **(a)** peroxide value (POV), **(b)***p-*anisidine value (PAV), **(c)** total oxidation (TOTOX) value, **(d)** integrated oxidation (INTOX) value, **(e)** conjugated diene value (CDV), and **(f)** conjugated triene value (CTV). Error bars represent SD of triplicate measurements. Samples A-C correspond to extra virgin olive oil (EVOO), and sample D corresponds to pomace olive oil (POO).

Fig. 3a presents the evolution of POV of olive oils during thermal oxidation at 180 °C. Initial POVs ranged from 5.37 meq/kg (sample D) to 15.3 meq/kg (sample C). After 16 h, POV increased moderately in sample D (6.7 meq/kg) but rose sharply in samples B and C, reaching 18.23 and 17.03 meq/kg, respectively. Sample A exhibited a non-linear trend, peaking at 16.97 meq/kg after 8 h before declining to 8.03 meq/kg. This reduction is attributed to the thermal instability of primary oxidation products, leading to decomposition of hydroperoxides into secondary volatile and non-volatile species (Plard et al., 2016).

In contrast, PAV, representing secondary oxidation products such as aldehydes and ketones, increased continuously in all samples throughout the heating (Fig. 3b). This behavior confirms that PAV is a more reliable indicator of advanced thermal oxidation stages than POV, as hydroperoxides are transient intermediates that decompose rapidly at 180 °C, while carbonyl compounds accumulate (Abdulkarim et al., 2007). Combined oxidation indices, TOTOX ([2 × POV] + PAV) and INTOX (POV + [2 × PAV]), provided an integrated estimate of the overall oxidation status of the oils (Fig. 3**c** and **d**). Sample D recorded the highest TOTOX and INTOX values, indicating the greatest overall susceptibility to oxidation, whereas the EVOO samples (particularly sample B) maintained lower total oxidation levels.

Both CDV and CTV increased progressively with heating time across all samples (Fig. 3**e** and **f**). Sample D (POO) exhibited the most substantial increase in CDV, rising from 15.63 to 47.27, whereas samples A, B, and C reached 31.10, 34.89, and 30.54, respectively. A similar trend was observed for CTV, further corroborating the greater extent of oxidation in the refined oil. These results are consistent with previous reports indicating that refined oils, which are depleted of natural antioxidants and phenolic compounds, are highly susceptible to accelerated conjugation reactions and the rapid degradation of polyunsaturated fatty acids (Abdulkarim et al., 2007; Gharby et al., 2016). The refining process specially bleaching and deodorization strips the oil protective pigments and endogenous stabilizers, thereby catalyzing the formation of conjugated systems (CDV and CTV) compared to the more stable EVOO matrix (Jabeur et al., 2016).

Overall, fluorescence spectroscopy revealed oxidation kinetics that are closely correlated with conventional oxidation analyses. The blue shift and intensity decay of the chlorophyll emission band at 675 nm directly reflect the progression of pigment demetallation and the concurrent accumulation of oxidation byproducts. The integrated fluorescence ratio and the resulting FRI thus provide a robust, non-destructive optical metric for the real-time monitoring of thermal oxidation in olive oils.

### 3.4 Lipid oxidation of olive oil upon UV exposure

**Fig. 4:**
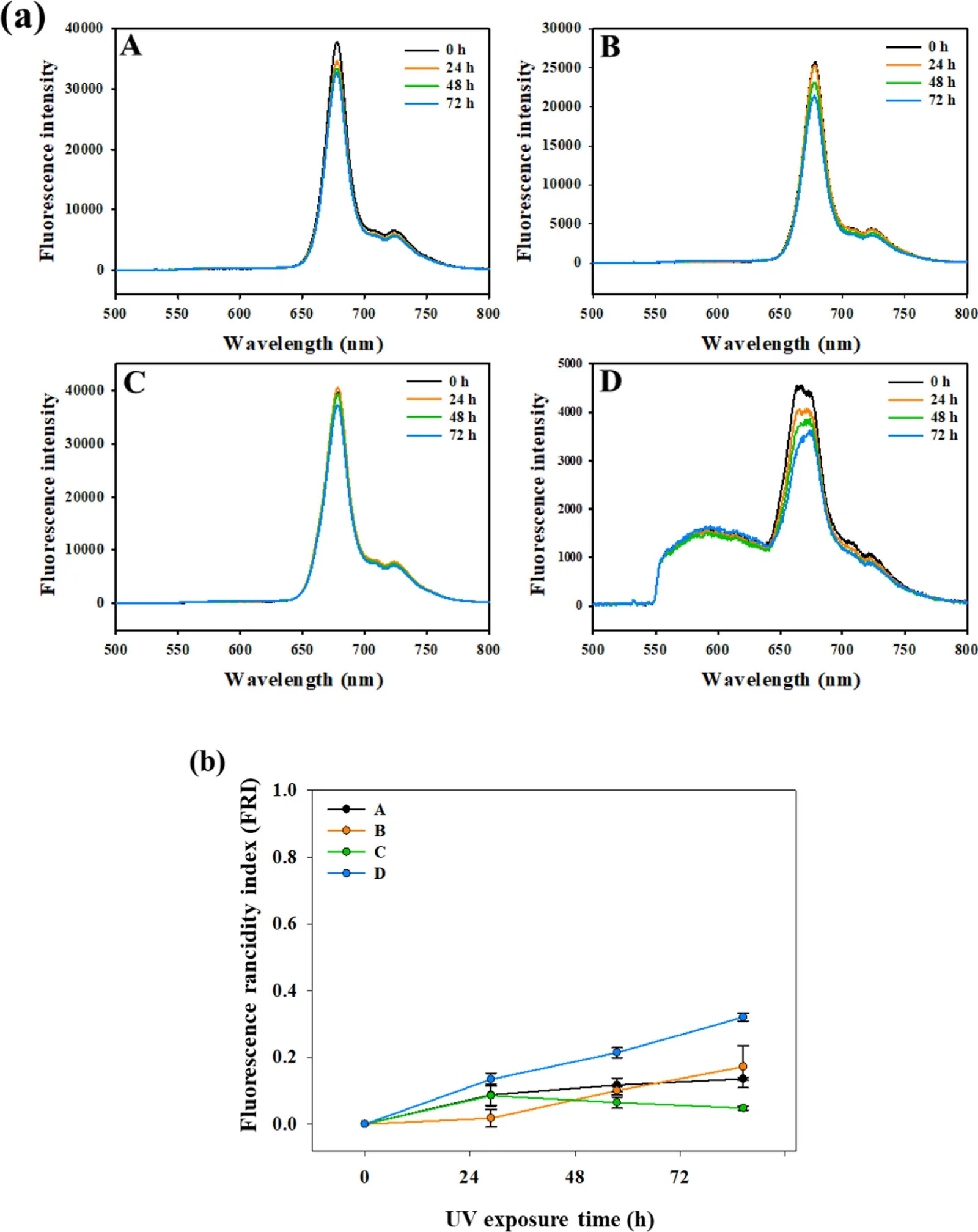
**(a)** Fluorescence emission spectra of olive oil samples exposed to UV irradiation for 0, 24, 48, and 72 h. **(b)** Time-dependent changes in fluorescence rancidity index (FRI) values calculated from the relative decrease in integrated fluorescence intensity over 640–725 nm. Samples A-C correspond to extra virgin olive oil (EVOO), and sample D corresponds to pomace olive oil (POO).

Fig. 4a illustrates the evolution of fluorescence spectra for oils exposed to UV radiation for up to 72 h. In contrast to thermal oxidation, the fluorescence spectral changes induced by UV exposure were less pronounced. Across all samples, the overall spectral profiles remained largely unchanged, although fluorescence intensity gradually decreased with increasing irradiation time. This behavior is consistent with a previous report indicating that photo-oxidation at ambient temperature generally proceeds more slowly than high temperature thermal oxidation because the latter provides significantly higher thermal energy, which accelerates radical chain reaction and pigment decomposition (Plard et al., 2016).

Despite the smaller magnitude of spectral variation, the FRI exhibited a steady, monotonic increase in all samples, confirming the progressive nature of photo-oxidative degradation (Fig. 4b). The high signal-to-noise and low variability observed among replicate measurements demonstrate the robustness of LIF system, highlighting its sensitivity to subtle oxidative changes that may be difficult to quantify by conventional chemical assays during early-stage photo-oxidation.

**Fig. 5:**
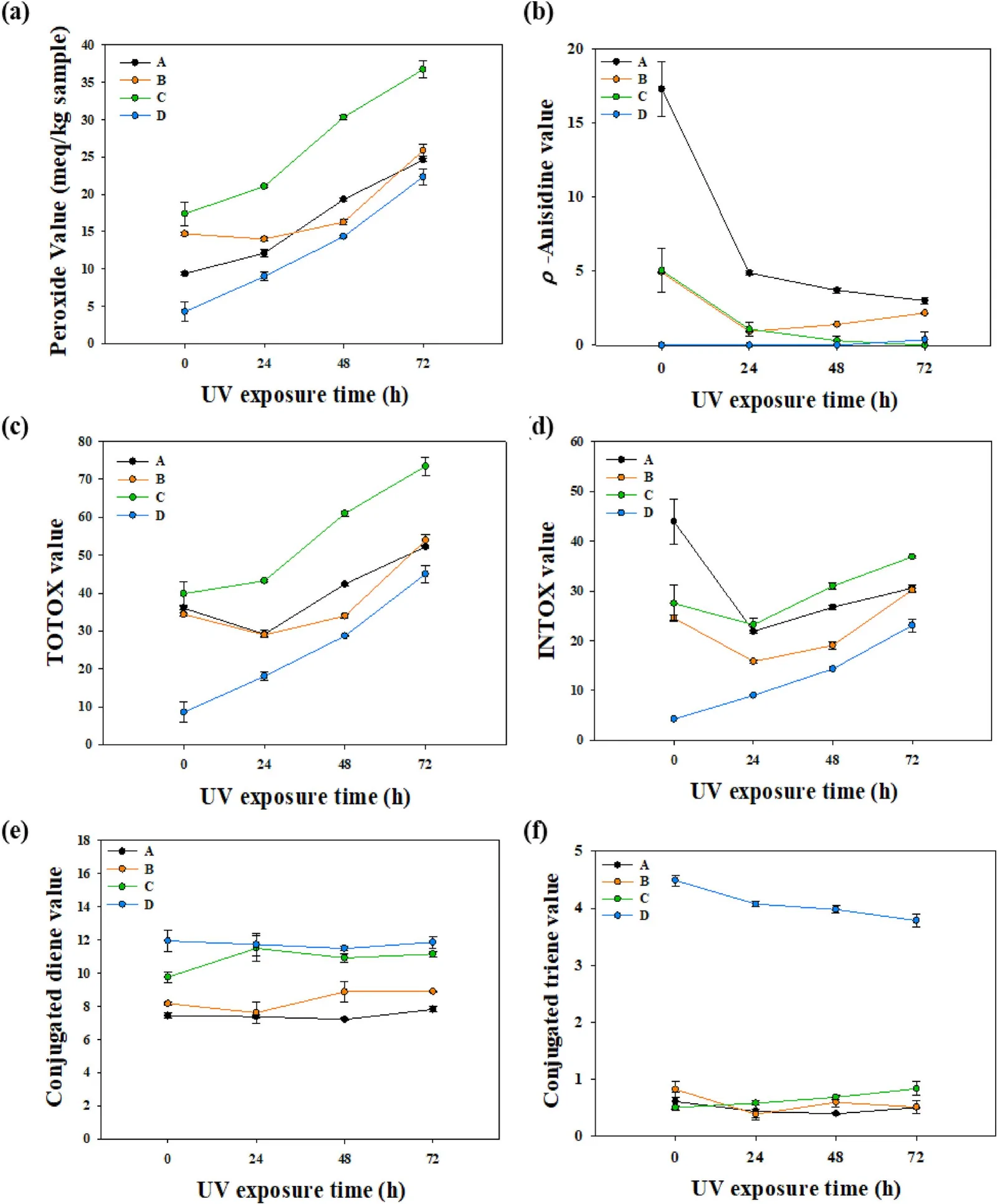
Changes in conventional oxidation indices of olive oil samples subjected to UV irradiation: **(a)** peroxide value (POV), **(b)***p*-anisidine value (PAV), **(c)** total oxidation (TOTOX) value, **(d)** integrated oxidation (INTOX) value, **(e)** conjugated diene value (CDV), and **(f)** conjugated triene value (CTV). Error bars represent SD of triplicate measurements. Samples A-C correspond to extra virgin olive oil (EVOO), and sample D corresponds to pomace olive oil (POO).

Fig. 5a displays the changes in POV during UV exposure. After 72 h, POV increased to 36.73, 25.87, 24.60, and 22.33 meq/kg for samples C, B, A, and D, respectively. Unlike the non-linear trends observed during thermal treatment, all samples exhibited a continuous increase in POV, indicating the sustained formation and relative stability of hydroperoxides under ambient photo-oxidative conditions. Conversely, the PAV decreased throughout the UV exposure period (Fig. 5b). This suggests the photodegradation or volatilization of secondary oxidation products. Due to these divergent trends, TOTOX followed the upward trajectory in POV **(**Fig. 5c), whereas INTOX did not show a clear increasing or decreasing trend during photo-oxidation (Fig. 5d).

CDV increased only slightly in EVOO samples (from 7.43 to 9.77 to 8.90–11.16), while remaining nearly constant in POO (∼11.90) after 72 h of UV exposure (Fig. 5e). Similarly, CTV remained stable across all samples, indicating limited isomerization and peroxidation under these mild photo-oxidative conditions (Fig. 5f). Previous study has demonstrated that light exposure accelerates oxidative deterioration of olive oil, particularly hydroperoxide formation as reflected by increased peroxide values (Houshia et al., 2019). In the present study, however, CTV remained relatively stable during the 72-h UV oxidation, suggesting that the applied photo-oxidative conditions produced only limited changes in conjugated triene-related structures. Mechanistically, this behavior can be attributed to the generation of singlet oxygen (^1^O₂) via energy transfer from photoexcited chlorophyll to ground-state triplet oxygen (^3^O₂) under light exposure (Fischer et al., 2013). The generated ^1^O₂ reacts preferentially with the double bonds of unsaturated fatty acids—particularly linoleic and linolenic acids—to form hydroperoxides. This process proceeds without the radical chain propagation typically associated with thermal oxidation (Min & Boff, 2002). Consequently, photo-oxidation is more selective and results in lower concentrations of secondary carbonyl compounds and conjugated systems compared to radical-mediated thermal oxidation (Poulli et al., 2009). Furthermore, the high oleic acid content in olive oil, characterized by a predominance of monounsaturated fatty acids, confers inherent resistance to conjugation and peroxidation under UV exposure. This compositional profile explains the minimal changes observed in CDV and CTV, especially when compared to oils rich in polyunsaturated fatty acids.

### 3.5 Simulated potato frying experiment

**Fig. 6:**
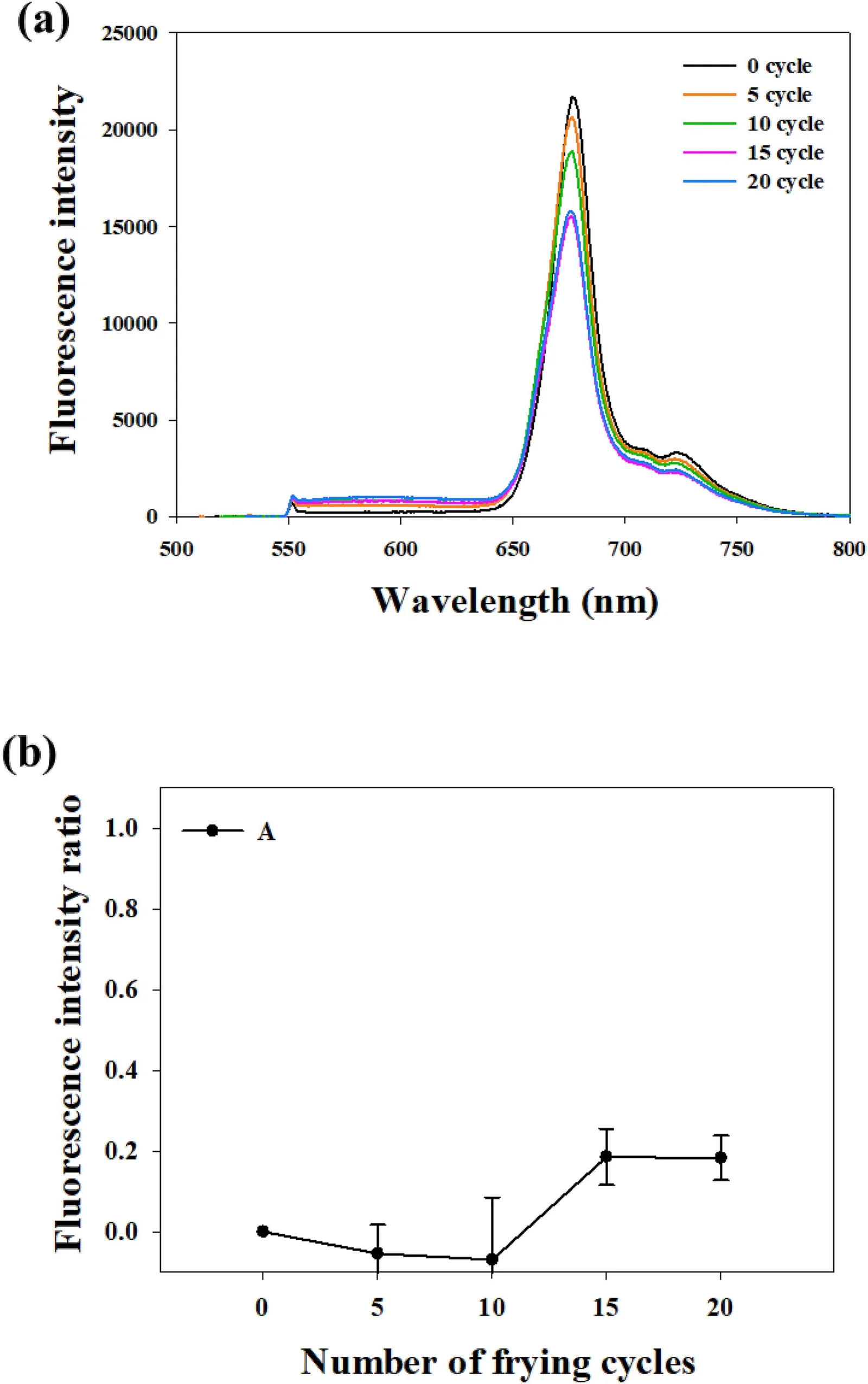
**(a)** Evolution of fluorescence spectra of olive oil samples during repeated frying cycles at 180 °C. **(b)** Corresponding fluorescence rancidity index (FRI) values calculated from the decreases of integrated fluorescence intensities over 640–725 nm, normalized to the initial value prior to frying. Error bars represent SD of triplicate measurements.

To simulate realistic culinary conditions, a model frying system was designed in which frozen potato strips were deep-fried at 180 °C for 3 min per cycle using a temperature-controlled electric fryer. Fluorescence spectra were recorded after each cycle to monitor the oxidative changes of the oils in situ. Sample A was selected as the representative EVOO for the potato frying model. Based on preliminary thermal oxidation data (Fig. 2), samples A, B, and C showed consistent oxidation kinetics. Sample A was specifically chosen due to its clear kinetic profile in POV trends and a lower initial oxidation level, ensuring a more accurate observation of oxidative progression throughout the frying cycles. Fig. 6(a) illustrates the spectral evolution of the olive oil sample (A) over up to 20 frying cycles, with the corresponding FRI values presented in Fig. 6(b). A progressive decrease in fluorescence intensity near 675 nm was observed with increasing frying cycles, consistent with the loss of chlorophyll-associated fluorescence during lipid oxidation. After 20 cycles, the FRI reached approximately 0.3, corresponding to a moderate level of oxidative degradation. Given that the cumulative heating time at 20 cycles was 60 min, the lower FRI values relative to those observed under continuous heating conditions (which reached higher FRI values in similar timeframes as seen in Fig. 2(b)) suggest that intermittent heating during frying modulates the oxidation rate. This effect is likely due to the cooling intervals between cycles and the presence of food moisture, which may reduce the cumulated thermal load and oxygen solubility within the matrix.

**Fig. 7:**
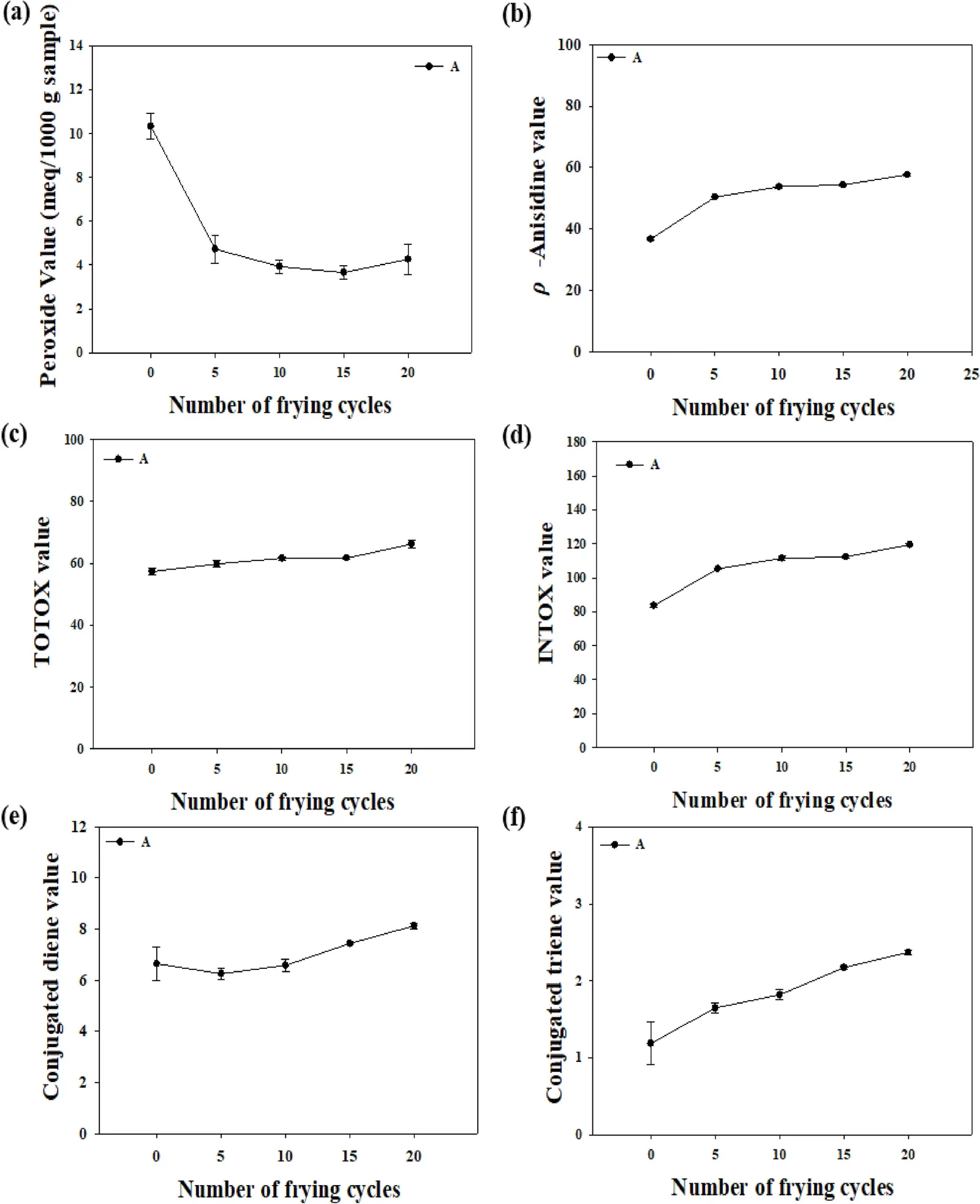
Changes in conventional oxidation indices of sample A (EVOO) during repeated potato frying at 180 °C: **(a)** peroxide value (POV), **(b)***p*-anisidine value (PAV), **(c)** total oxidation (TOTOX) value, **(d)** integrated oxidation (INTOX) value, **(e)** conjugated diene value (CDV), and **(f)** conjugated triene value (CTV). Error bars represent SD of triplicate measurements.

Fig. 7(a) shows the changes in POV for sample A during repeated frying cycles. POV gradually decreased as frying progressed, reflecting the rapid thermal decomposition of hydroperoxides at 180 °C, which outpaces their formation rate under high-heat, intermittent conditions. In contrast, the PAV increased sharply from 37 to 58 (Fig. 7(b)), indicating the steady accumulation of volatile and non-volatile aldehydic secondary products, a common feature of prolonged deep-frying (Houhoula et al., 2002). Both the TOTOX and INTOX values increased steadily throughout the experiment (Fig. 7**c** and **d)**). However, INTOX showed a steeper trajectory, highlighting the dominant role of secondary oxidation products in overall oil deterioration during frying. Juncos et al. (2024) demonstrated that the INTOX index provides superior sensitivity during advanced oxidation stages, particularly when peroxides become thermally unstable and decompose into stable carbonyl compounds.

Fig. 7 shows the gradual increases in CDV and CTV over 20 frying cycles. Collectively, both the fluorescence-based parameters and the conventional oxidation indices demonstrate that oxidation during simulated frying progresses more slowly than under continuous thermal treatment. Nevertheless, repetitive heating resulted in cumulative oxidative degradation, characterized by hydroperoxide decomposition, aldehyde accumulation, as well as the formation of conjugated bond systems. These results confirm that fluorescence spectroscopy effectively captures complex oxidation dynamics under realistic frying conditions. By monitoring these changes via the FRI, this approach provides a robust, non-invasive platform for real-time monitoring of oil stability during both industrial and culinary frying operations.

Similarly, other extra virgin olive oils (Samples B and C) followed a comparable gradual trend of FRI increase. In contrast, Sample D (Pomace Olive Oil) exhibited a much faster increase in FRI due to its lower initial pigment concentration and lack of natural antioxidants. Although these findings demonstrate the potential of the FRI, it is important to acknowledge that the simulated potato frying experiment was primarily conducted using sample A as a representative extra virgin olive oil. While the consistency of oxidation kinetics across extra virgin samples was confirmed through preliminary thermal oxidation data, the specific interactions between the food matrix and different oil compositions warrant further investigation. Future research should therefore encompass a wider variety of oil types, including those with divergent pigment profiles and initial antioxidant capacities, to further validate the universality and robustness of the FRI. Additionally, expanding the dataset size will be essential in future studies to improve the statistical power of the correlation models and to refine the predictive performance of the index under diverse industrial and culinary environments.

### 3.6 Correlation analysis

Pearson correlation analysis was performed to assess the associations between the FRI and conventional lipid oxidation indices under thermal and UV oxidation conditions (Table 1). Under thermal oxidation, the FRI showed strong positive correlations with PAV (*r* = 0.92, 95% CI: 0.77–0.97, *p* < 0.001), TOTOX (*r* = 0.91, 95% CI: 0.75–0.97, *p* < 0.001), INTOX (*r* = 0.92, 95% CI: 0.77–0.97, *p* < 0.001), and CDV (*r* = 0.88, 95% CI: 0.67–0.96, *p* < 0.001). A moderate positive correlation was observed with CTV (*r* = 0.62, 95% CI: 0.17–0.85, *p* = 0.01), whereas no significant correlation was found with POV (*r* = 0.09, 95% CI: −0.43–0.56, *p* = 0.75).

**Table 1:** Pearson correlations between the fluorescence rancidity index (FRI) and conventional lipid oxidation indices under thermal and UV oxidation conditions.

	Thermal oxidation				UV oxidation		
Oxidation index	Pearson's r	95% CI for r	*p*-value		Pearson's r	95% CI for r	p-value
POV	0.09	[−0.43, 0.56]	0.75		0.00	[−0.49, 0.50]	0.99
PAV	0.92	[0.77, 0.97]	<0.001		−0.38	[−0.74, 0.14]	0.15
TOTOX	0.91	[0.75, 0.97]	<0.001		0.19	[−0.34, 0.63]	0.48
INTOX	0.92	[0.77, 0.97]	<0.001		−0.31	[−0.70, 0.22]	0.24
CDV	0.88	[0.67, 0.96]	<0.001		0.31	[−0.22, 0.70]	0.24
CTV	0.62	[0.17, 0.85]	0.01		0.42	[−0.10, 0.76]	0.11

Pearson correlation analyses were performed separately for thermal and UV oxidation, each using 16 oil–time condition means (four oil samples × four time points). Triplicate measurements for each oil–time condition were averaged before analysis. Shown are Pearson correlation coefficients (r), two-sided *p*-values, and 95% confidence intervals (CIs) are presented. Statistical significance was defined as *p* < 0.05. POV, peroxide value; PAV, *ρ-*anisidine value; TOTOX, total oxidation value; INTOX, integrated oxidation value; CDV, conjugated diene value; CTV, conjugated triene value.

These findings suggest that the FRI is more closely associated with secondary oxidation and the formation of conjugated lipid structures than with the immediate concentration of hydroperoxides. The weak or negative correlation between the FRI and POV is attributed to the instability of hydroperoxides, which decompose into secondary oxidation products during advanced oxidation (Guzmán et al., 2015). Thus, POV does not necessarily reflect pigment degradation. In contrast, PAV captures aldehydic secondary oxidation products and conjugated-diene-related measurements reflect structural changes in oxidized unsaturated lipids.

A different pattern emerged under UV oxidation: none of the correlations between FRI and the conventional oxidation indices reached statistical significance. This disparity indicates that the relationship between fluorescence changes and conventional oxidation indices is highly dependent on the oxidation pathway. Light exposure can simultaneously induce lipid photooxidation and alter endogenous pigments, while conventional indices reflect distinct oxidation products and stages. For example, light exposure has been linked to increased POV and concurrent losses of chlorophyll and carotenoid pigments (Houshia et al., 2019). Thus, lack of significant correlations under UV oxidation does not necessarily indicate an absence of oxidative or fluorescence changes; but rather, that FRI and conventional indices did not vary in a sufficiently coupled linear manner.

Importantly, the present correlation and regression analyses demonstrate associations within this dataset, but do not validate FRI as a quantitative prediction model. Further studies with larger independent datasets, and appropriate validation needed to establish the FRI's predictive performance, robustness, and generalizability across diverse oils and oxidation conditions.

## 4 Conclusion

This study introduces a mechanistically grounded fluorescence-based analytical framework for monitoring lipid oxidation in olive oils, moving beyond qualitative spectral observation toward a quantitatively defined FRI. Unlike previous fluorescence approaches that relied primarily on multivariate discrimination or empirical modeling, the proposed FRI is derived from the fundamental physicochemical coupling between chlorophyll degradation and lipid oxidation pathways. This enables direct and more intuitive interpretation of oxidative progression. While FRI showed strong correlation with several secondary and conjugation-related oxidation indices during thermal oxidation, these associations were not significant under UV oxidation. The robustness, reproducibility, and spectrally normalized formulation of the FRI facilitate cross-sample comparison without the need for extensive sample preparation or hazardous chemical reagents. These characteristics position fluorescence spectroscopy not merely as a complementary technique, but as a viable, green analytical platform for the real-time, non-destructive monitoring of oxidative stability in both laboratory settings and industrial frying environments.

Although these results demonstrate the feasibility of FRI for monitoring oxidative changes in the olive oils studied, the limited sample diversity should be considered when assessing its broader applicability. Because FRI is relies in part chlorophyll-associated fluorescence, variations in the initial content and composition of chlorophylls and their derivatives may influence both baseline fluorescence and its response to oxidation. Future studies should assess a larger and more diverse range of oils encompassing broader pigment compositions and processing conditions to determine the robustness and generalizability of the FRI and to establish appropriate quantitative thresholds for different oxidation stages.

## CRediT authorship contribution statement

**Yubeen Lee:** Writing – original draft, Methodology, Investigation, Formal analysis. **Jee Won Lee:** Writing – original draft, Methodology, Investigation, Formal analysis. **Jinhee Kang:** Writing – original draft, Investigation, Formal analysis. **Won Young Jeong:** Writing – original draft, Data curation. **Hyung Min Kim:** Writing – review & editing, Validation, Supervision, Project administration. **Jee-Young Imm:** Writing – review & editing, Validation, Supervision, Project administration.

## Declaration of competing interest

The authors declare that they have no known competing financial interests or personal relationships that could have appeared to influence the work reported in this paper.

## Data Availability

Data will be made available on request.
