# Supplementary material for: Mechanistically derived fluorescence rancidity index for real-time monitoring of olive oil oxidation under thermal, photo, and frying conditions
Source: Food Chem X. 2026 Sep 1;39:104381. doi: 10.1016/j.fochx.2026.104381 (PMC13599668; doi:10.1016/j.fochx.2026.104381)
Supplement: Supplementary file 1 [file mmc1.docx]

**Supplementary Data**

**Table S1.** Simple linear regression analysis between FRI and conventional lipid oxidation indices.

| **Oxidation index** | **Slope** | **Intercept** | **R²** | **n** |
| --- | --- | --- | --- | --- |
| **Thermal** | | | | |
| **POV** | 0.00728 | 0.4387 | 0.007 | 16 |
| **PAV** | 0.00427 | -0.0645 | 0.838 | 16 |
| **TOTOX** | 0.00417 | -0.1441 | 0.827 | 16 |
| **INTOX** | 0.00214 | -0.0898 | 0.838 | 16 |
| **CDV** | 0.02771 | -0.1125 | 0.768 | 16 |
| **CTV** | 0.06563 | 0.1343 | 0.380 | 16 |
| **UV** | | | | |
| **POV** | 2.30 × 10⁻⁵ | 0.0931 | 5.30 × 10⁻⁶ | 16 |
| **PAV** | -0.00787 | 0.1156 | 0.143 | 16 |
| **TOTOX** | 0.00105 | 0.0518 | 0.036 | 16 |
| **INTOX** | -0.00219 | 0.1119 | 0.097 | 16 |
| **CDV** | 0.01496 | -0.0504 | 0.097 | 16 |
| **CTV** | 0.02355 | 0.0594 | 0.174 | 16 |

**Fig S1. Fluorescence spectra of toluene (a) and chlorophyll standard (b)**

**Fig S2.** Photograph of a compact laser-induced fluorescence (LIF) prototype illustrating the feasibility of a portable laser-excitation and fiber-coupled fluorescence detection system for in situ monitoring of olive oil oxidation.
